# Can Alkaline Hydrolysis of γ-HCH Serve as a Model Reaction to Study Its Aerobic Enzymatic Dehydrochlorination by LinA?

**DOI:** 10.3390/ijms20235955

**Published:** 2019-11-26

**Authors:** Suraj Kannath, Paweł Adamczyk, Langping Wu, Hans H. Richnow, Agnieszka Dybala-Defratyka

**Affiliations:** 1 Institute of Applied Radiation Chemistry, Faculty of Chemistry, Lodz University of Technology, Zeromskiego 112, 90-924 Lodz, Poland; surajkannath91@gmail.com (S.K.); pawel_adamczyk@yahoo.com (P.A.); 2 Department of Isotope Biogeochemistry, Helmholtz Centre for Environmental Research-UFZ, Permoserstraße 15, 04318 Leipzig, Germany; langping.wu@utoronto.ca (L.W.); hans.richnow@ufz.de (H.H.R.); 3 Departments of Civil & Mineral Engineering, University of Toronto, 35 St. George St., Toronto, ON M5S 1A4, Canada

**Keywords:** kinetic isotope effects, explicit solvation, elimination reaction, QM/MM, density functional theory, path integral, hexachlorocyclohexanes

## Abstract

Hexachlorocyclohexane (HCH) isomers constitute a group of persistent organic pollutants. Their mass production and treatment have led to a global environmental problem that continues to this day. The characterization of modes of degradation of HCH by isotope fractionation is a current challenge. Multi isotope fractionation analysis provides a concept to characterize the nature of enzymatic and chemical transformation reactions. The understanding of the kinetic isotope effects (KIE) on bond cleavage reaction contributes to analyses of the mechanism of chemical and enzymatic reactions. Herein, carbon, chlorine, and hydrogen kinetic isotope effects are measured and predicted for the dehydrochlorination reaction of γ-HCH promoted by the hydroxyl ion in aqueous solution. Quantum mechanical (QM) microsolvation with an implicit solvation model and path integral formalism in combination with free-energy perturbation and umbrella sampling (PI-FEP/UM) and quantum mechanical/molecular mechanical QM/MM potentials for including solvent effects as well as calculating isotope effects are used and analyzed with respect to their performance in reproducing measured values. Reaction characterization is discussed based on the magnitudes of obtained isotope effects. The comparative analysis between the chemical dehydrochlorination of γ-HCH in aqueous media and catalyzed reaction by dehydrochlorinase, LinA is presented and discussed. Based on the values of isotope effects, these two processes seem to occur via the same net mechanism.

## 1. Introduction

Hexachlorocyclohexane (HCH) isomers are persistent organic pollutants that pose a serious threat to the state of the natural environment, including, of course, health and life of humans and other live beings [1,2,3,4]. The use of technical grade HCH and large amounts of waste containing α, β, and δ-HCH isomers due to the production of Lindane (γ-HCH) has resulted in the worldwide spread of HCH today. α-, β- and γ-HCH were listed in the Stockholm Convention of persistent organic pollutants (POPs) [5]. According to estimates 1.7–4.8 million tons of HCH are still present widely in nature [6]. Isotope fractionation concepts have been used to elucidate transformation reactions of HCH in field studies [7,8] and diagnostically to characterize transformation reactions even in food webs [9,10].

For the interpretation of isotope effects in the environments the understanding of the nature of KIE is a current challenge in environmental science and modelling of experimental data is needed for deeper understanding the transition states of the bond cleavage reaction [11].

Kinetic isotope effects (KIEs) can provide information about the structure of the transition state and in this way bring us closer to determining the mechanism by which a given reaction may occur. Unfortunately, very often due to the complexity of reactions they do not provide unambiguous answers. At least two independent tools have been used to study the fate of HCH isomers so far. One of them is compound-specific isotope analysis (CSIA), a technique that measures the ratios of stable isotopes (e.g., carbon, chlorine, nitrogen, hydrogen) for a given compound, as opposed to a specific atomic position within a molecule. CSIA can be considered as an average isotopic composition coming from all atomic positions of a selected element present in the studied molecule. Thus, isotope effect at any of the atomic positions becomes diluted due to the presence of the same element at other positions within the same molecule [12]. It is compared to international standards and as a result allows us to assess the degree and source of pollutants’ degradation [12,13]. γ-HCH metabolism by LinA has been very recently studied using this technique by Schilling et al. [14]. They investigated enzyme specificity of carbon and hydrogen KIEs associated with the dehydrochlorination of γ-HCH by two variants of lindane dehydrochlorinase, LinA1 and LinA2 and found only small variations in isotope effects between these two proteins. The authors measured quite substantial carbon isotope effects and rather small hydrogen isotope effects. The other tool is based on the theoretical KIEs prediction. The theoretical insight from some of us, based on full enzymatic model [15] allowed to characterize mechanistic pathways viable for the transformation of γ-HCH by LinA and eventually led to the carbon, chlorine, and hydrogen KIEs prediction [16,17]. Some discrepancies, such as much larger predicted hydrogen KIE, have been found in the data coming from these two approaches, thus opening room for debate. 

**Scheme 1:**
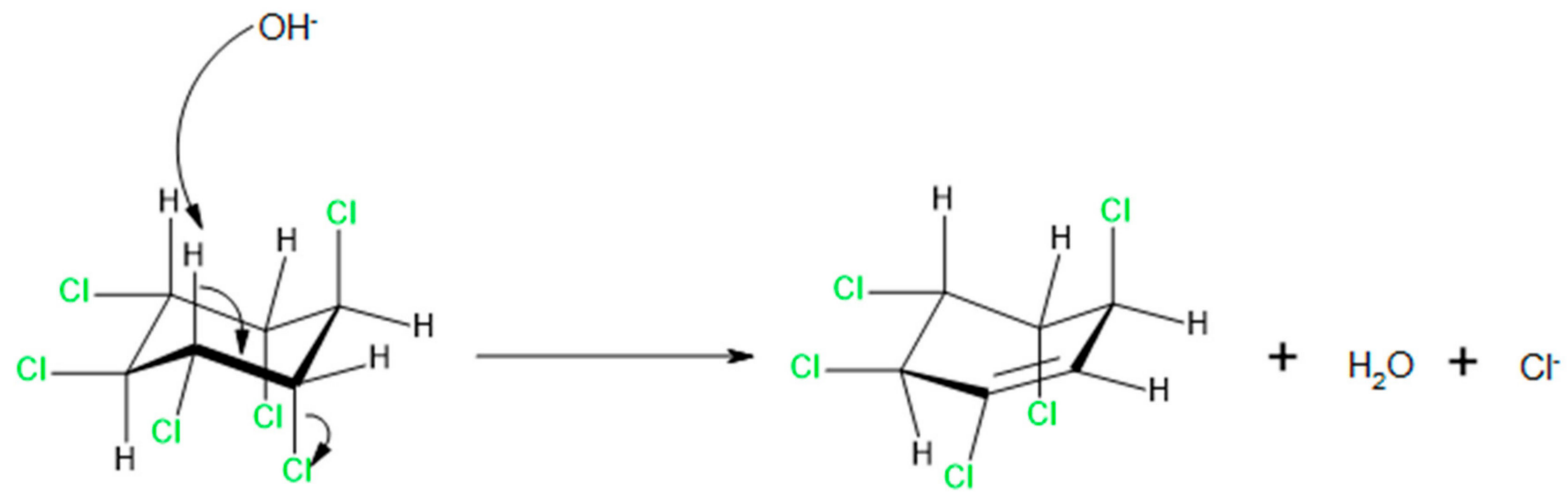
Dehydrochlorination of γ-HCH by hydroxyl anion.

In order to study the role of an enzyme in performing a specific reaction it is worth studying the formal identical reaction, but without the enzyme presence, in aqueous solution. Such an attempt has already been undertaken [18] but using very limited approaches with respect to model size and thus intermolecular interactions for both enzymatic and non-enzymatic transformation of γ-HCH. In aqueous solution under neutral conditions, degradation of γ-HCH is an extremely slow reaction. The measured rate constant was 8.9 × 10^−9^ s^−1^ [19]. Under alkaline conditions, however, the reaction can speed up by almost 5 orders of magnitude [20]. In the current work, we carried out the dehydrochlorination reaction of γ-HCH under alkaline conditions (Scheme 1) using both experimental and theoretical approaches. The main goal of the present study was three-fold. First, we wanted to find a theoretical model that would allow to reproduce and to improve interpretation of the experimental kinetic data. Second, we aimed to provide a theoretical description of dehydrochlorination of γ-HCH occurring in solution under different conditions (neutral vs alkaline). To this end, we analyzed various size microsolvation models used to mimic the solvation of the reacting species as well as a full explicit solvent model. Specific interactions with respect to the number of water molecules directly interacting with the solutes as well as their nature (donating or accepting protons) are also discussed. Finally, we utilized the obtained isotopic data for the non-enzymatic reaction and reported earlier results for the enzymatic transformation of γ-HCH to discuss the effect of an enzyme with respect to the molecular mechanism via which the first bond cleavage takes place.

## 2. Results and Discussion

In this study we assumed that under alkaline conditions in aqueous solution the γ-HCH molecule is attacked by the hydroxyl ion. Previously, we utilized neutral conditions and modeled the elimination reaction having a water molecule as a base abstracting proton from the substrate molecule [18]. As kinetic isotope effects for the transformation of γ-HCH in aqueous solution were not available by that time, we did not analyze them in very much detail. Now, based on the previously constructed models we predicted position-specific C, Cl, and H KIEs using the level of theory selected in the present work (Table S1) and calculated bulk (average) isotope effects for each studied element (Table 1). Here, we showed them only for one solvation model (attacking water molecule was microsolvated by two other explicit water molecules and the bulk solvent effects for the whole system were included using the polarizable continuum model, PCM) as in the present study (all details on performed herein calculations are given in the Materials and Methods section including proper references of the applied methods and techniques). To our surprise, predicted C and Cl KIEs turned out to be in very good agreement with the experimental values. Hydrogen KIE, however, is not in accord; it is much more pronounced than the measured value which might be an indication of the inappropriateness of the model, as expected, or insensitiveness of heavy atom KIEs to the nature of the base in this reaction as compared to H KIE. These aspects will be further discussed in the subsequent sections of the article. 

**Figure 1:**
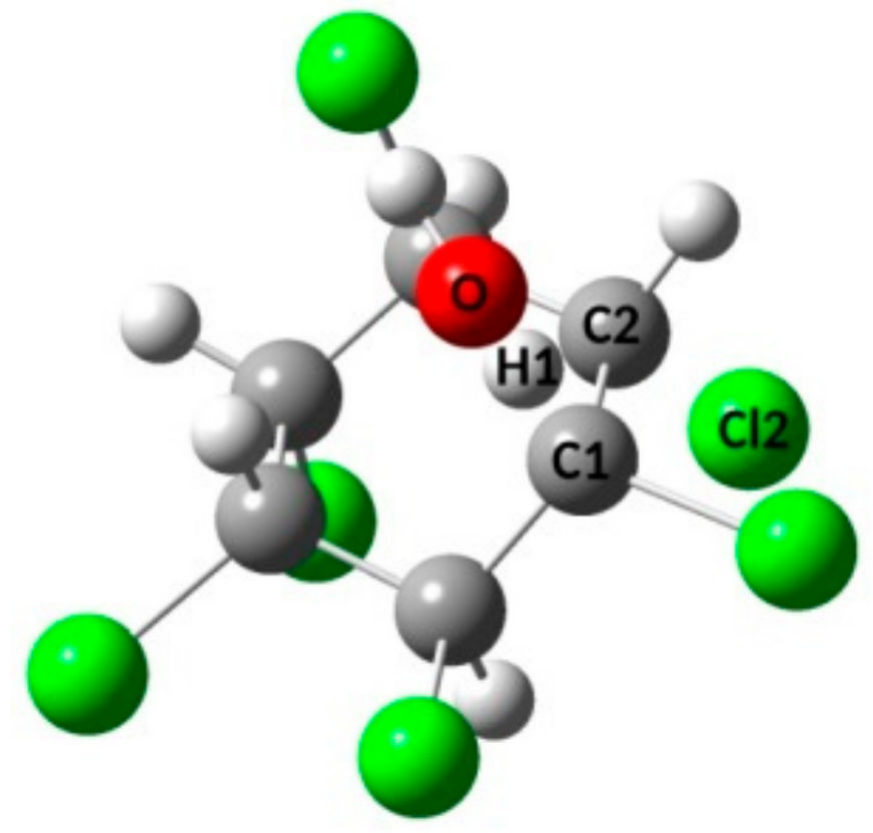
Atom numbering of the key atomic positions in the reaction between γ-HCH isomer and the hydroxyl ion.

γ-HCH has two feasible positions at which the OH^−^ ion can attack the hydrogen atom. However, since they are symmetrical to each other and lead to the same products only one was taken into account (Figure 1). We constructed the two-dimensional ωB97xD/6-311+G(d,p)/PCM free energy surface by including the proton (H1) transfer variable and the carbon-chlorine bond (C2-Cl2) cleavage as reaction coordinates (Figure S1). Only one transition structure comprising these two chemical events was located. Next, this initial bare model was enlarged by placing a different number of water molecules near the attacking OH^−^ ion and the chloride leaving group.

It has been previously demonstrated that the coordination number for the OH^−^ ion in aqueous solution might be up to 3÷4 molecules [21] whereas for the chloride anion it was rather 6 [22,23] in the first solvation shell. In our models we surrounded OH^−^ by maximum 4 and Cl^−^ by 6 water molecules. We noticed that in the case of both considered attack positions cleavage of the C-Cl bond was barely advanced at the transition state. Furthermore, the Merz-Singh-Kollman charge [24,25] localized on the leaving Cl atom is very small, around only −0.2e at the transition state (Table S6a,b). This might suggest that placing water molecules at the OH^−^ side may play the larger role than at the Cl side. However, at the products state when the C-Cl bond is fully broken, the charge is close to −1e and then the chloride ion readily interacts with surrounding water molecules. This would rather be a base for building models in which both the hydroxyl ion and the chlorine substituent undergoing elimination are properly solvated. In order to consider these two hypotheses the following microsolvation models were constructed: (1) with 1 water molecule near OH^−^ (1W_OH); (2) with 1 water molecule near both OH^−^ and Cl (1W_OH-1W_Cl); (3) with 2 water molecules near OH^−^ (2W_OH); (4) with 2 water molecules near both OH^−^ and Cl (2W_OH-2W_Cl); (5) with 3 water molecules near OH^−^ (3W_OH); (6) with 3 water molecules near both OH^−^ and Cl (3W_OH-3W_Cl); (7) with 3 water molecules near OH^−^ and 4 near Cl (3W_OH-4W_Cl); (8) with 3 water molecules near OH^−^ and 5 near Cl (3W_OH-5W_Cl); (9) with 3 water molecules near OH^−^ and 6 near Cl (3W_OH-6W_Cl); (10) with 4 water molecules near OH^−^ (4W_OH); (11) with 4 water molecules near OH^−^ and 4 near Cl (4W_OH-4W_Cl); (12) with 4 water molecules near OH^−^ and 5 near Cl (4W_OH-5W_Cl); (13) with 4 water molecules near OH^−^ and 6 near Cl (4W_OH-6W_Cl), and (14) with 5 water molecules near OH^−^ (5W_OH) (Figure S7). A total of 15 cluster models including the bare model were built. For each of them the ωB97xD/6-311+G(d,p)/PCM free energy surface and the kinetic isotope effects for all carbon, chlorine, and hydrogen positions within the γ-HCH molecule were calculated (Tables S2 and S4). Based on this position-specific data we calculated the average KIE for each element and derived isotopic enrichment factor, ε using Equation (1) to compare our prediction with the measured values of KIE. For clarity we split the KIEs data into two groups; one collecting results for the models containing water molecules only near the hydroxyl group, and the other containing values for the models having OH^−^ and Cl^−^ ions solvated (Figure S2). The general observation is that none of the constructed models allowed to reproduce any of the experimental isotope effects. However, the predicted magnitudes for carbon fractionation were only slightly underestimated as compared to chlorine or hydrogen fractionation. Between two sets of models, the one in which only the hydroxyl group was microsolvated performed slightly better and, starting from the 3W_OH model, the average carbon isotope effect converged. Magnitude-wise, inclusion of the chlorine first solvation shell does not seem to affect the predicted values of isotope effects. The similar observation can also be made for two other elements, chlorine and hydrogen. In the case of the chlorine isotope effect, the predictions are also lower than the measured values, but the difference between theory and experiment is larger than in the case of carbon isotope effect. This could mean that the calculated chlorine KIEs at the reactive (Cl2) position should be at least twice as large. The predicted value averaged over all microsolvation models was 1.0027 ± 0.0005. Hydrogen KIEs tend to be hugely overestimated by the both sets of microsolvation models. The effect on the abstracted proton (H1) is 5.1 ± 0.1, which again demonstrates that the performance of all models in this respect was very similar. However, apparently the hydrogen kinetic isotope effect that could be derived from the measured bulk H effects for the reacting H1 position should be smaller at least by half. 

**Figure 2:**
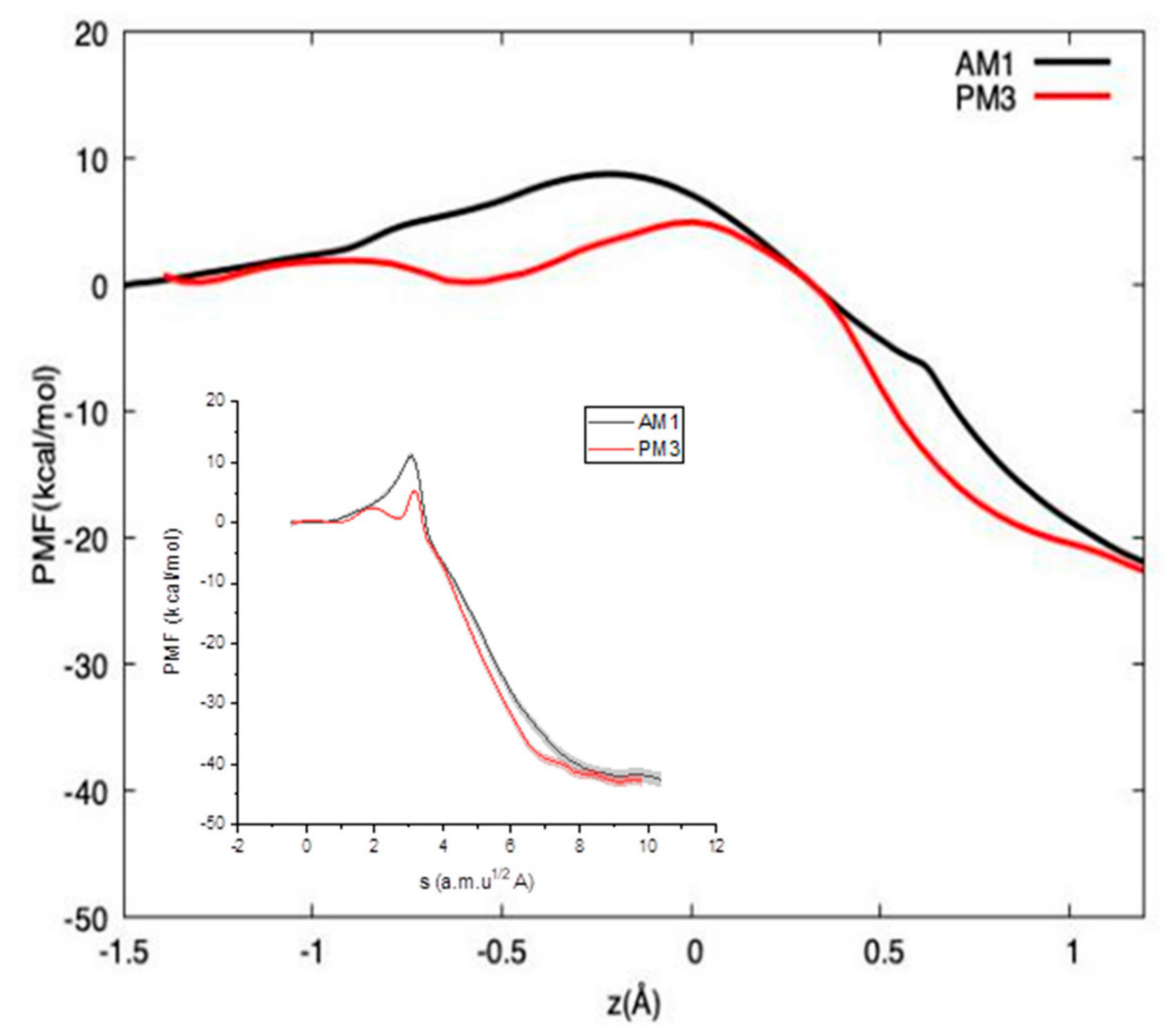
Free energy profile as a function of reaction coordinate (z = [C1–H1] − [H1^…^O] in Å) resulted from umbrella sampling simulations using the quantum mechanical/molecular mechanical (QM/MM) potential in CHARMM. Inset contains the path CV resulted from the string calculations in Amber using QM(PM3)/MM (red curve) and QM(AM1)/MM (black curve) potentials. The error bars (shadowed area) present the 95% confidence interval in the free energy.

The reasons for the aforementioned failure of microsolvation models and semiclassical treatment of isotope effects can be the following: (i) insufficient model with respect to how the solvation of the system is represented; (ii) inadequate theory used to predict KIEs (lack of inclusion of nuclear quantum effects, lack of consideration of ensemble of TSs and instead taking into account a single TS structure, and lack of proper sampling of solvent). In order to address at least part of these deficiencies we applied the path integral formalism combined with free-energy perturbation-umbrella sampling simulation (PI-FEP/UM) methodology combined with the QM/MM potential. Using the QM/MM description of the studied system the specific solute-solvent interactions and the explicit solvent presence in bulk as well as an ensemble of located transition state structures were taken into account. We performed QM/MM MD simulations within which the QM(PM3)/MM and QM(AM1)/MM potentials of mean force (PMF) for the elimination of one H/Cl pair from the HCH molecule by OH^−^ were computed using the umbrella sampling technique and the string method. Within the first approach the reaction coordinate was defined in such a way that only variables related to the proton abstraction part were taken into account. To reduce the cost of simulations the variable associated with the carbon-chlorine bond cleavage was not included as that would require 2D umbrella sampling simulations. Being, however, aware of the importance of its inclusion in the reaction coordinate definition we additionally used the adaptive string method implemented recently in Amber by Zinovjev and Tuñón [26] to include more variables and to check this way whether the reaction pathways resulted from these two approaches are going to be consistent. The resulting free energy profiles from both of these methods are shown in Figure 2. The free energy cost to reach the transition state using the PM3 Hamiltonian is 5 and 6.2 ± 0.7 kcal mol^−1^ from the umbrella sampling and string calculations, respectively. Having AM1 as a method to describe the QM region the free energy of activation increased to 8.4 and 11.1 ± 0.6 kcal mol^−1^, respectively. As the free energy barriers resulted from the semiempirical treatment of the QM atoms are either a bit lower or higher as compared to the barriers obtained for the microsolvation models (Table S2) we made an attempt to correct them using a higher level of theory. After correction, the estimated barriers are 12.7 and 11.9 kcal mol^−1^ for the models originated from the QM(PM3)/MM and QM(AM1)/MM simulations, respectively. 

Simulations using both techniques, umbrella sampling and string, allowed us to arrive at the same conclusions with respect to the reaction mechanism. They were also consistent with the results obtained for the QM microsolvation models indicating that the reaction between γ-HCH and OH^−^ is a concerted elimination pathway. All transition states located using the QM microsolvation models have characteristics with the proton transfer significantly advanced and chlorine departure negligibly progressed. From the structural point of view it might indicate a E1cb-like character of the transition state, however, the atomic charges analysis did not show the formation of carbocation at the C1 position from which proton is abstracted (Table S6a,b). In contrast, the QM/MM models resulted in transition states for which proton abstraction was a bit less advanced and carbon-chlorine bond cleavage was more advanced (Figure S7). Having confirmed the mechanism, we utilized the trajectories resulting from the umbrella sampling simulations for subsequent path integral calculations. Using this approach, the nuclear quantum mechanical free energy corrections such as the free energy difference and individual free energy contribution for different isotopic substitution (Figures S3 and S4) as well as kinetic isotope effects at the reactive positions within the HCH molecule for different number of beads (replicas of the quantized part of the system) (Table S5) were calculated. For both semiempirical Hamiltonians used to treat the QM region of the system 64 beads were sufficient to obtain the converged results. The inclusion of quantum effects led to the decrease of free energy of activation (total quantum effects, ΔΔG_qm_^≠^) of 2.2 and 3.1 for H, and 1.6 and 2.0 for D, for QM(AM1)/MM and QM(PM3)/MM, respectively (Figures S5 and S6). In the case of carbon, the effect on the free energy of activation was similar; however, the effect caused by heavier isotopolog of carbon (^13^C) was as expected smaller than the one for deuterium (0.2 for ^13^C vs 0.6 or 1.1 kcal mol^−1^ for ^2^H) (Figures S5 and S6). For chlorine the total quantum effect was 2.0 and 1.7 kcal mol^−1^ for AM1 and PM3 treatment of the QM region and no difference within the accuracy of the potential and the method was found for the ^37^Cl isotopolog.

As calculations of KIEs for all atomic positions (reactive and non-reactive) within a substrate molecule using the PI-FEP/UM methodology would be too expansive we limited them only to the reactive (primary) positions. The secondary (non-reactive) positions, although often involved in coupling between the reaction mode and other modes in a system, should not be affected by effects such as tunneling or recrossing, in particular heavy atom positions like carbon or chlorine. Therefore, omitting them should not in principle change the data qualitatively. Besides, a very small intervention of quantum effects for these elements has been already pointed out. The computed average contribution coming from these positions based on the data collected in Table S4 is 1.0029, 1.0008, and 1.0156 for C, Cl, and H, respectively. We, however, decided to use bulk secondary effects originating from those microsolvation models that first of all resulted in the closest agreement with the experimental data and secondly provided very similar geometries of the located transition states to the ones obtained from the QM/MM potentials. The former requirement is met by 3W_OH and 4W_OH-4W_Cl models, and the latter one by the bare model (Figure S7).

Next, the isotope effects from those models along with the isotope effects predicted for reactive positions using the QM/MM models were used to estimate enrichment factors (ε) that we treated as bulk isotope effects predicted using the QM/MM models (Table 1). Evidently, the QM(PM3)/MM potential provided poorer agreement with the experimental values than QM(AM1)/MM. Interestingly, the values resulted from using different microsolvation models as a source of secondary positions are quite similar. Altogether, we see that bulk carbon and chlorine isotope effects are quite insensitive to the nature of the base abstracting the proton from the substrate molecule. However, none of the models allowed us to predict hydrogen isotope effect correctly. In all cases the predicted magnitude of the primary kinetic isotope effect must be overestimated since all bulk H effects are hugely overestimated. In order to assess the performance of each methodology we listed all primary carbon, chlorine, and hydrogen KIEs in Table 2. Indeed, all models that are considered to provide the most accurate predictions in this study resulted in H KIE of 4÷5, regardless the nature of the base and the progress of the proton transfer between the substrate and the base (Figure S7 and Table S3). In QM microsolvation models the proton transfer is more advanced than in the case of the bare and QM/MM models. The PI calculations based on the QM(AM1)/MM trajectories provided however the values of isotope effects that are in the closest agreement with the experiment. Carbon KIE is almost the same, chlorine KIE is slightly underestimated, whereas hydrogen KIE is heavily overestimated. The reason for this could be the low accuracy of the used potential (AM1) and lack of secondary H KIEs predicted directly using the PI approach. Assuming that the primary H KIE is around 4÷5, in order to have bulk H at the level of −160‰, the majority of accompanying secondary H KIE should be inverse (less than unity). In contrast, Cl KIE differed a lot among the models; for instance, the bare model provided the smallest effect whereas the one in which water plays the role of a base provided the largest. 

We finally analyzed how isotope effects on the elimination reaction of H/Cl pair from γ-HCH by OH^−^ compare with the isotopic data obtained experimentally and computationally for enzymatically catalyzed elimination by LinA (Table 3). 

The magnitudes of isotope effects obtained for enzymatically catalyzed elimination reaction are very similar to the ones resulted from the alkaline hydrolysis. Although two different experimental studies (entries denoted by a and b superscripts) slightly differ in the magnitude of bulk carbon isotope effect, this difference might reflect only minor changes in the transition state geometry. It is worth noting that basically the values of isotope effects obtained for enzymatic and non-enzymatic reaction are very close to each other, which would mean that in both cases the underlying reaction mechanism is the same and only some subtle differences in the transition state geometries should be expected (i.e., the extent of proton transfer or the progress of carbon-chlorine bond cleavage). In particular that multi- instead of single-element approach was used in studying both processes and isotopic fractionation measured for all three (C, Cl, and H) agree quite well. Therefore, the effect of enzyme on the H/Cl pair elimination from the γ-HCH isomer in the overall transformation would lie in the rate enhancement rather than in any mechanism alterations. If dehydrochlorination takes place in the active site of LinA the H1 proton is abstracted by neutral His73 which interacts strongly with neighboring Asp25. The latter residue provides negative charge to the reaction and its role ranges from being in hydrogen bonding contact with the base [27] to abstracting the proton from His73 once the elimination of H/Cl pair from the substrate molecule is completed to keep the base residue singly-protonated [15]. A picture drawn from the predicted magnitudes of isotope effects on hydrolysis in aqueous solution as well as on enzymatic elimination along with the detailed structural information coming from theoretical studies leads to quite different, however, interesting observations. Under alkaline conditions the net charge of the reaction is exactly the same as in the active site, however, the base abstracting proton carries the negative charge. If water is the base, on the other hand, it becomes positively charged (protonated) upon reaching the transition state as in the case of His73. Inspection of transition state geometries clearly demonstrates that this different protonation state of involved species has a strong effect on the progress of specific reaction variables such as proton abstraction from substrate and the carbon-chlorine bond elongation. As a result, proton abstraction is advanced to a similar extent in neutral hydrolysis and enzymatically catalyzed reaction whereas elimination of the leaving group (Cl^−^) and the carbon-carbon double bond formation resembles rather the reaction taking place under alkaline conditions (Figure S7, Table S7). 

## 3. Materials and Methods 

### 3.1. Free Energy Surfaces for the QM Microsolvation Models

As a first step of our study we constructed free energy surfaces for the H/Cl elimination reaction promoted by the hydroxyl ion using the ωB97xD/6-311+G(d,p) [28,29,30,31,32,33] level of theory implemented in the Gaussian G09 software [34]. In order to include the bulk effect of solvation, IEFPCM continuum solvent model [35] was used. Specific interactions between the solute molecules and the solvent were taken into account by constructing various models in which different number of explicit water molecules microsolvating ionic species in the studied reaction was present. All geometry optimizations were carried out using very tight convergence criteria and ultra-fine integrals. Transition states of modeled reactions were located using the Berny algorithm [36,37], then Intrinsic Reaction Coordinate (IRC) [38] protocol was applied to investigate reactions profiles, which end point geometries were optimized to either reactants or products. Vibrational analysis accompanied every optimization procedure to confirm that obtained geometries corresponded to either local minimum (no imaginary frequencies found) or first-order saddle point (exactly one imaginary frequency) on the free energy surfaces and to calculate position specific kinetic isotope effects using the Bigeleisen equation [39,40], implemented in the ISOEFF program [41]. Next, all carbon (C KIE = ^12^k/^13^k), chlorine (Cl KIE = ^35^k/^37^k), and hydrogen (H KIE = ^1^k/^2^k) position specific KIEs for a given element, were averaged (KIE_av_) and so-called bulk enrichment factors for the whole compound (γ-HCH) were calculated using the formula:

(1) $$\varepsilon =\left(1-KIE_{av}\right)\cdot 1000‰$$

Free energies in solution were calculated using zero-point energies and thermal contributions to the Gibbs free energy computed using the same level of theory.

Atomic charges were calculated using the Merz-Singh-Kollman scheme [24,25].

### 3.2. PI-FEP/UM

The other method used to predict KIEs on the reaction taking place in an aqueous solution was the path integral formalism combined with free-energy perturbation-umbrella sampling simulation (PI-FEP/UM) [42]. The method is one of the most efficient approaches for treating nuclear quantum effects and is especially well known for providing accurate results for KIEs on reactions in solutions and in enzymes with the environment explicitly present in a model. All path integral simulations were carried out using the CHARMM package [43]. Within the first umbrella sampling [44,45] part of the entire PI-FEP/UM simulations the QM region of the model containing the entire HCH molecule and the hydroxyl ion was modeled using the semi-empirical PM3 [46,47] and AM1 [48] methods and the solvent region was represented by 1080 TIP3P [49] water molecules. Stochastic boundary conditions were used to control behavior of the solvent. The solvated system was then heated up to 298 K for 20 ps and brought to an equilibrium during the next 20 ps. The reaction coordinate was defined as a combination of the C1-H1 bond cleavage and the H1-O bond formation (Scheme 1). 14 regions (so-called windows) were needed to cover the entire reaction range. Each window was equilibrated for 100 ps and the data collection was carried out for the subsequent 200 ps. Potential of mean force (PMF) for the reaction was extracted using Weight Histogram Analysis Method (WHAM) [50,51]. Path integral simulations were subsequently performed and KIEs were calculated using free energy perturbation [52] method using the classical trajectories resulted from the PMF generation. The quantum contributions were estimated using the bisection sampling (BQCP) [42,53] method which is extended form of QCP [54,55,56] method. In order to perform the PI calculations, selected atoms were quantized: for C1 and H KIEs only the C1, H1, O atoms were quantized and for C2 and Cl2 KIEs all the QM atoms were quantized with various numbers of beads (P equal to 8, 16, 32, 64). Around 8000 and 7000 classical configurations from QM(PM3)/MM and QM(AM1)/MM simulations, respectively were used for the PI simulations for which each classical step consisted of 10 path integral steps. 

In order to either confirm or disregard the reaction mechanism resulted from the umbrella sampling simulations we also performed the calculations of the minimum free energy path by using the string method [57] and defining a collective coordinate as the advance of the system along this path to obtain the reaction PMF. For this purpose, we applied the latest implementation of the string method in the Amber simulation package [58] (the adaptive string method, ASM [26]). Details of these simulations are provided in Supplementary Materials.

### 3.3. Determination of the Experimental Isotope Enrichment Factors of γ-HCH

Hydrolysis of γ-HCH was carried out in 100 mM NaOH-H_3_BO_3_ buffer at 30 °C (pH 10,) as batch experiments. The enzymatic degradation of γ-HCH by LinA was performed in TRIS buffer (pH 7.5) at 10 °C and 120 rpm shaking. The reactions were stopped at different time intervals and the remaining γ-HCH was extracted for concentration and stable carbon, hydrogen and chlorine isotope analysis. The analytical methods have been described in detail elsewhere [9,10] and basic information is provided in Supplementary Materials.

## 4. Conclusions

In the present work, we studied kinetic isotope effects associated with dehydrochlorination of γ-HCH by the hydroxyl ion in aqueous solution. Different models of the reaction and two methodologies; QM microsolvation and transition state theory and QM/MM description combined with the PI-FEP/UM calculations allowed to reproduce measured isotope effects only in part. Full explicit presence of the solvent within QM/MM potentials and PI treatment of isotope effects led to quite good agreement with the experimental values of carbon and chlorine isotope effects. However, the prediction of hydrogen isotope effect caused the major problem as all performed computations led to a large overestimation of the effect. This in part could be due to the too low quality of the applied potentials (PM3 or AM1). Unfortunately, the calculations using QM(DFT)/MM would be too expensive with respect to the computational resources required to perform. Another possible source of the poorer agreement between theoretically and experimentally determined H KIEs could come from the treatment of secondary isotope effects. The outcome from the calculations might improve if all hydrogen isotope effects (primary and secondary) are directly computed instead of being assumed as they were in the case for secondary (non-reactive) positions. Again, such an approach could not have been used due to the cost of PI calculations for so many (six) atomic positions within a molecule. 

Nevertheless, between two QM/MM models QM(AM1)/MM provided much better agreement with the experimental values. It also allowed for exploring the interactions of the solute molecules with the surrounding water molecules. It was found that the reaction started with the hydroxyl ion solvated by 4 water molecules in the direct neighborhood, then this number decreases to 3 at transition state and finally drops down to 1 or 0 at products state. All these water molecules play the role of the proton donor. In the case of the ionic species being formed during the reaction (Cl^−^) the observed trend is opposite; the reaction starts with no direct interaction between the chlorine substituent and the solvent molecules and ends with 4÷5 at the products. Such change in the pattern of direct interactions between the solute and the solvent available only when full explicit solvation model is used can at least partially help to explain some deficiencies found for the QM microsolvation models where a number of such interactions was fixed throughout all calculations involving a specific model. 

Analysis of three different kinetic isotope effects measured and predicted for enzymatic and non-enzymatic elimination revealed that both processes occur via the same net mechanism. These observations could possibly constitute some suggestions of using the alkaline hydrolysis reaction and as a model reaction to study the aerobic transformation of HCH isomers that undergo LinA-catalyzed metabolism. Such multi-element approach applied in the environmental studies allows to improve identification and differentiation of transformation pathways in the environment. As one element isotopic analysis may not be sufficient for these tasks as its fractionation can be similar even for two distinctive metabolic pathways more sophisticated approaches are sought. Before they are, however, applied on a larger scale (i.e., in the field to detect groundwater pollutants) appropriate knowledge on multi isotope effects accompanying a reaction of interest should be gained. It is a demanding task from both experimental and computational points of view; hence, studies combining these two approaches should be continued in order to collect more data on various compounds and the transformation processes they undergo.

## Figures and Tables

**Table 1:** Measured and predicted carbon, chlorine, and hydrogen bulk kinetic isotope effects, ε (‰) on dehydrochlorination of γ-HCH.

Model	ε_C_	ε_Cl_	ε_H_
exp ^a^	−7.0 ± 0.5	−2.0 ± 0.2	−162 ± 26
H_2_O_2W ^b^	−6.5	−1.5	−526
3W_OH	−5.7	−0.8	−692
4W_OH−4W_Cl	−6.0	−0.9	−680
QM(AM1)/MM ^c^	−7.5 (−6.7)	−1.4 (−1.2)	−481 (−463)
QM(PM3)/MM ^c^	−3.6 (−2.8)	−1.0 (−0.8)	−756 (−738)

^a^ Compound-specific C, Cl, and H isotope enrichment factors were also measured in this study on hydrolysis of γ-HCH carried out at 30 °C and pH 10 as described in detail in Supplementary Materials. ^b^ coordinates of models taken from [18] and recalculated using the ωB97xD/6-311+G(d,p) level of theory. ^c^ values in parentheses are obtained using the bare model of a source of secondary position-specific isotope effects.

**Table 2:** Primary carbon, chlorine, and hydrogen kinetic isotope effects on H/Cl pair elimination from the HCH molecule by OH^−^ using selected QM microsolvation and QM/MM models.

Model	C1 KIE	C2 KIE	Cl KIE	H1 KIE
H_2_O_2W ^a^	1.0182	1.0190	1.0081	4.0
Bare model	1.0113	1.0068	1.0018	5.1
3W_OH	1.0178	1.0113	1.0029	5.1
4W_OH-4W_Cl	1.0190	1.0144	1.0036	5.0
QM(AM1)/MM	1.0197 ± 0.0039	1.0204 ± 0.0010	1.0063 ± 0.0006	3.8 ± 0.2
QM(PM3)/MM	1.0083 ± 0.0019	1.0086 ± 0.0082	1.0037 ± 0.0020	5.5 ± 0.4

^a^ coordinates of models taken from [18] and recalculated using the ωB97xD/6-311+G(d,p)/PCM level of theory.

**Table 3:** Bulk carbon, chlorine, and hydrogen kinetic isotope effects, ε (‰) on H/Cl pair elimination from the HCH molecule catalyzed by LinA.

Reaction	ε_C_	ε_Cl_	ε_H_
[14]^a^	−8.1 ± 0.3 (−8.3 ± 0.2)	n/d	−122 ± 6 (−160 ± 6)
This study	−5.3 ± 0.8	−1.8 ± 0.4	−119 ± 18
[16,17] ^b^	−5.0	−0.7	−633

^a^ values in parentheses denote the reaction catalyzed by different protein variant. ^b^ calculated using predicted primary KIE and assuming no effect for all secondary positions.

## Supplementary Materials

Supplementary materials can be found at https://www.mdpi.com/1422-0067/20/23/5955/s1.

## Abbreviations

KIE	Kinetic isotope effect
PI	Path integral
PI-FEP/UM	Path integral combined with free energy perturbation and umbrella sampling
QM	Quantum mechanical
QM/MM	Quantum mechanics/molecular mechanics
HCH	Hexachlorocyclohexane
PMF	Potential of Mean Force
CSIA	Compound-specific isotope analysis
PCM	Polarizable continuum model
MD	Molecular dynamics
