# Supplementary material for: Can Alkaline Hydrolysis of γ-HCH Serve as a Model Reaction to Study Its Aerobic Enzymatic Dehydrochlorination by LinA?
Source: Int J Mol Sci. 2019 Nov 26;20(23):5955. doi: 10.3390/ijms20235955 (PMC6929183; doi:10.3390/ijms20235955)
Supplement: Supplementary file 1 [file ijms-20-05955-s001.pdf]

Supplementary Material

For

Can Alkaline Hydrolysis of γ-HCH Serve as a Model Reaction to Study Its Aerobic Enzymatic Dehydrochlorination by LinA?

Suraj Kannath,[1] Paweł Adamczyk,[1] Langping Wu,[2,3] Hans H. Richnow,[2]

and Agnieszka Dybala-Defratyka[1*]

*[1] Institute of Applied Radiation Chemistry, Faculty of Chemistry,
Lodz University of Technology, Zeromskiego 116, 90-924 Lodz, Poland*

[2] Department of Isotope Biogeochemistry, Helmholtz Centre for Environmental Research-UFZ, Permoserstraße 15, 04318 Leipzig, Germany

[3] Departments of Civil & Mineral Engineering, University of Toronto, 35 St. George St. Toronto, ON M5S 1A4, Canada

e-mail: agnieszka.dybala-defratyka@p.lodz.pl

[*] To whom correspondence should be addressed

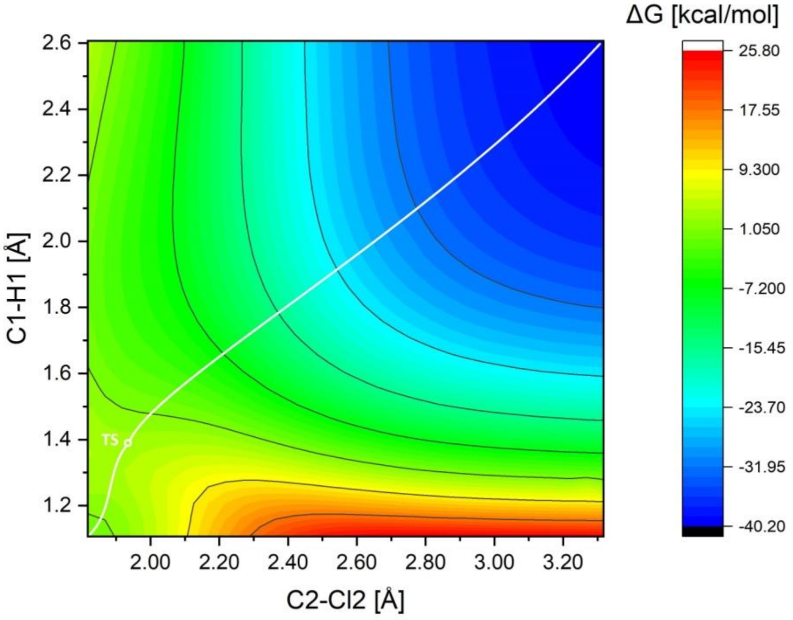

Figure S1. 2D free energy surface for the reaction between γ-HCH and OH^- in aqueous solution.

Table S1. Carbon, chlorine and hydrogen position specific kinetic isotope effects on the reaction of γ-HCH with water molecule resulted from the QM microsolvation model obtained at the ωB97-XD/6-311+G(d,p) /PCM level of theory. Primary kinetic isotope effects are shown in bold.

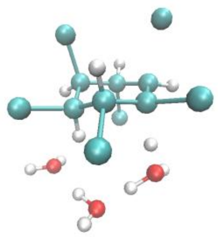

$^{12}C/^{13}C$	$^{35}Cl/^{37}Cl$	$^{1}H/^{2}H$
0.9992	1.0004	1.0382
1.0025	1.0001	1.0086
**1.0182**	1.0005	1.1001
**1.0190**	**1.0081**	1.0088
1.0010	1.0000	1.0310
0.9994	1.0000	**3.9670**

Table S2. Free energies of activation ($\Delta G^{\neq}$, kcal mol^{-1}) and reaction (ΔG_R, kcal mol^{-1}), reaction coordinate mode (*i*freq, cm^{-1}) for dehydrochlorination of γ-HCH obtained at the wB97XD/6-311+G(d,p) /PCM level of theory.

Model	$\Delta G^{\neq}$	ΔG_R	*i*freq
**Bare**	0.7	-46.4	1216
**1W_OH**	4.8	-42.9	1242
**1W_OH_1W_Cl**	4.5	-41.0	1256
**2W_OH**	7.6	-38.6	1029
**2W_OH_2W_Cl**	7.5	-34.6	1209
**3W_OH**	7.0	-37.3	986
**3W_OH_3W_Cl**	5.6	-34.1	1187
**3W_OH_4W_Cl**	9.2	-29.3	1030
**3W_OH_5W_Cl**	9.0	-30.0	1134
**4W_OH**	13.4	-42.9	937
**4W_OH_4W_Cl**	13.5	-28.4	903
**4W_OH_5W_Cl**	8.9	-31.9	1174
**4W_OH_6W_Cl**	7.7	-37.5	1176
**5W_OH**	8.6	-36.9	1008

Table S3. Key geometry parameters for reactants, transition state and products obtained for the reaction between γ-HCH and OH^-.

Model	Reactants				Transition state				Product			
	C-H (Å)	C-Cl (Å)	H-O (Å)	C-C (Å)	C-H (Å)	C-Cl (Å)	H-O (Å)	C-C (Å)	C-H (Å)	C-Cl (Å)	H-O (Å)	C-C (Å)
**Bare**	1.11	1.82	2.01	1.53	1.32	1.87	1.29	1.50	3.16	4.23	0.96	1.33
**1W_OH**	1.11	1.82	1.92	1.53	1.39	1.89	1.22	1.49	3.39	4.18	0.96	1.33
**1W_OH_1W_Cl**	1.11	1.82	1.92	1.53	1.38	1.90	1.24	1.49	2.80	3.65	0.96	1.33
**2W_OH**	1.11	1.81	1.96	1.52	1.46	1.90	1.17	1.48	3.54	4.15	0.96	1.33
**2W_OH_2W_Cl**	1.11	1.82	1.95	1.53	1.41	1.93	1.20	1.48	3.51	4.13	0.96	1.33
**3W_OH**	1.10	1.81	2.06	1.53	1.46	1.91	1.16	1.48	2.80	4.24	0.96	1.33
**3W_OH_3W_Cl**	1.10	1.82	2.04	1.53	1.41	1.93	1.20	1.48	2.64	3.85	0.96	1.33
**3W_OH_4W_Cl**	1.11	1.82	1.95	1.53	1.46	1.93	1.16	1.47	3.32	4.02	0.97	1.33
**3W_OH_5W_Cl**	1.11	1.82	1.89	1.52	1.43	1.92	1.19	1.47	3.42	3.78	0.97	1.33
**4W_OH**	1.11	1.82	1.91	1.53	1.47	1.91	1.15	1.48	3.46	3.78	0.96	1.33
**4W_OH_4W_Cl**	1.09	1.81	2.44	1.53	1.49	1.96	1.15	1.46	3.57	3.59	0.97	1.33
**4W_OH_5W_Cl**	1.10	1.82	2.00	1.53	1.41	1.91	1.20	1.48	2.85	3.76	0.96	1.33
**4W_OH_6W_Cl**	1.11	1.82	1.95	1.52	1.40	1.91	1.21	1.48	3.09	4.47	0.96	1.33

**5W_OH**	1.11	1.82	1.91	1.52	1.46	1.91	1.16	1.48	3.60	4.33	0.97	1.33
**QM(PM3)/MM**	1.17±0.02	1.82±0.02	2.40±0.05	1.50±0.00	1.36±0.04	1.87±0.05	1.30±0.04	1.48±0.01	2.09±0.10	2.91±0.08	0.95±0.02	1.35±0.02
**QM(AM1)/MM**	1.27±0.23	1.80±0.07	2.34±0.12	1.51±0.03	1.30±0.04	1.84±0.04	1.36±0.08	1.50±0.05	2.15±0.04	2.95±0.08	1.00±0.01	1.35±0.02

Table S4. All carbon, chlorine and hydrogen position specific kinetic isotope effects on the reaction of γ-HCH with OH^- resulted from the QM microsolvation models. Primary kinetic isotope effects are shown in bold.

	$^{12}C/^{13}C$						$^{35}Cl/^{37}Cl$						$^{1}H/^{2}H$					
**Atom number**	**C_1**	**C_2**	C_3	C_4	C_5	C_6	Cl_1	**Cl_2**	Cl_3	Cl_4	Cl_5	Cl_6	**H_1**	H_2	H_3	H_4	H_5	H_6
**Bare**	**1.0113**	**1.0068**	1.0005	0.9985	0.9991	1.0020	1.0007	**1.0018**	1.0000	0.9999	0.9999	1.0005	**5.1200**	1.0191	0.9982	0.9915	1.0354	0.9137
**1W_OH**	**1.0143**	**1.0083**	1.0004	0.9999	1.0006	1.0035	1.0008	**1.0022**	1.0001	1.0000	1.0001	1.0004	**4.9900**	1.0199	0.9998	1.0012	1.0423	0.9890
**1W_OH_1W_Cl**	**1.0132**	**1.0086**	1.0003	0.9996	1.0002	1.0031	1.0006	**1.0022**	1.0000	0.9999	1.0001	1.0004	**4.9100**	1.0196	1.0056	0.9974	1.0391	1.0080
**2W_OH**	**1.0171**	**1.0108**	1.0002	0.9997	1.0004	1.0041	1.0009	**1.0027**	1.0003	0.9999	1.0001	1.0006	**5.0800**	1.0236	0.9887	1.0004	1.0470	1.0114
**2W_OH_2W_Cl**	**1.0143**	**1.0107**	1.0006	0.9996	0.9999	1.0035	1.0007	**1.0028**	1.0000	0.9999	1.0000	1.0006	**4.9400**	1.0241	1.0092	0.9986	1.0500	1.0153
**3W_OH**	**1.0178**	**1.0113**	1.0006	0.9997	1.0005	1.0041	1.0010	**1.0029**	1.0002	1.0000	1.0001	1.0009	**5.0900**	1.0275	0.9957	0.9959	1.0654	0.9794
**3W_OH_3W_Cl**	**1.0141**	**1.0104**	1.0002	0.9992	1.0002	1.0028	1.0007	**1.0027**	1.0001	0.9999	1.0000	1.0007	**5.2100**	1.0505	0.9958	0.9958	1.0390	1.0053
**3W_OH_4W_Cl**	**1.0166**	**1.0116**	1.0002	0.9999	1.0001	1.0034	1.0007	**1.0030**	1.0003	1.0001	1.0001	1.0007	**5.0700**	1.0330	1.0014	0.9992	1.0521	1.0013

**3W_OH_5W_Cl**	**1.0154**	**1.0104**	1.0002	0.9994	1.0001	1.0029	1.0007	**1.0018**	1.0001	0.9998	0.9999	1.0005	**5.1600**	1.0268	0.9923	0.9921	1.0430	0.9959
**4W_OH**	**1.0182**	**1.0113**	1.0003	0.9997	1.0004	1.0036	1.0009	**1.0029**	1.0001	1.0001	1.0001	1.0007	**5.0100**	1.0317	1.0081	0.9968	1.0576	0.9965
**4W_OH_4W_Cl**	**1.0190**	**1.0144**	1.0001	0.9993	1.0002	1.0032	1.0010	**1.0036**	1.0003	0.9999	1.0000	1.0004	**4.9700**	1.0193	1.0038	1.0011	1.0466	1.0377
**4W_OH_5W_Cl**	**1.0145**	**1.0101**	1.0005	1.0000	1.0005	1.0036	1.0007	**1.0027**	1.0002	1.0000	1.0000	1.0006	**5.2300**	1.0223	0.9904	1.0047	1.0457	1.0172
**4W_OH_6W_Cl**	**1.0148**	**1.0091**	1.0005	0.9996	0.9999	1.0034	1.0007	**1.0023**	1.0001	0.9999	1.0000	1.0006	**5.0000**	1.0149	1.0002	1.0037	1.0443	1.0222
**5W_OH**	**1.0171**	**1.0110**	1.0007	0.9996	1.0002	1.0035	1.0008	**1.0028**	1.0001	1.0001	1.0001	1.0006	**5.0800**	1.0334	0.9922	0.9921	1.0541	0.9979

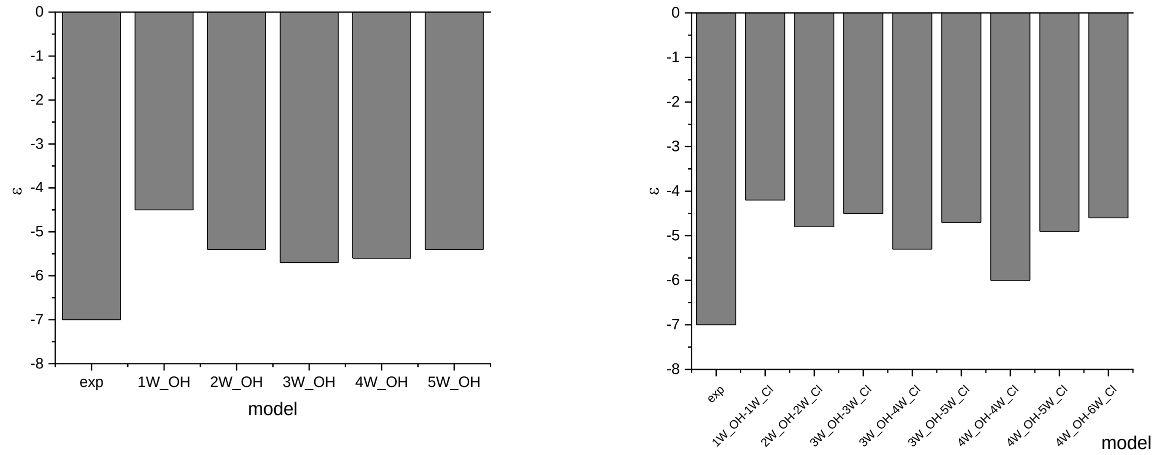

εC

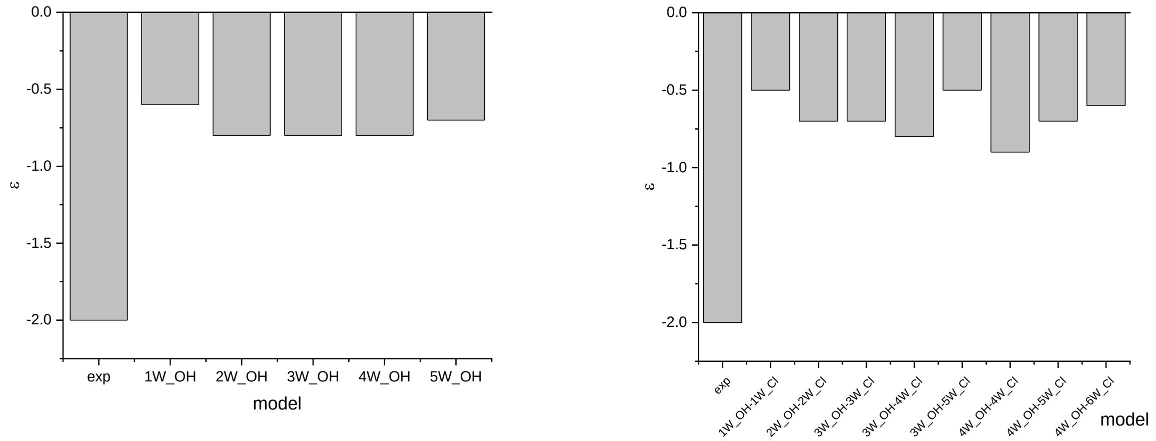

εCl

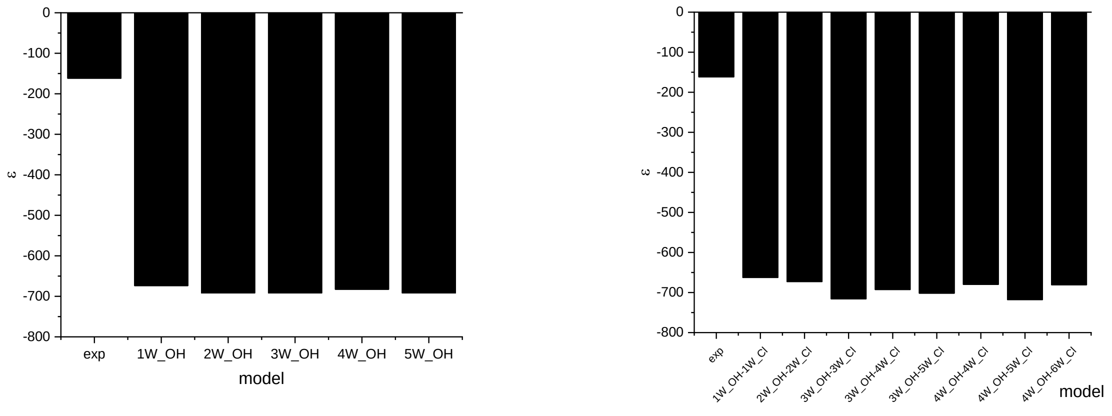

εH

Figure S2. Bulk carbon, chlorine, and hydrogen kinetic isotope effects on dehydrochlorination of γ-HCH by OH^- calculated at the ωB97xD/6-311+G(d,p)/PCM level of theory.

Table S5. Primary carbon, chlorine, and hydrogen kinetic isotope effects on the H/Cl pair elimination from the HCH molecule by OH^- using the PI-FEP/UM method, different number of beads, and the AM1 and PM3 Hamiltonians for the QM region treatment in QM/MM models.

QM method	No. of beads	PI-FEP/UM			
		C_1	H_1	C_2	Cl_2
**PM3**	8	1.0051±0.0037	4.45±0.18		
	16	1.0050±0.0031	5.02±0.25		
	32	1.0096±0.0045	5.53±0.67	1.0106±0.0083	1.0041±0.0004
	64	1.0083±0.0019	5.47±0.39	1.0086±0.0082	1.0037±0.0020
**AM1**	32	1.0192±0.0053	3.62±0.19	1.0261±0.0061	1.0060±0.0004
	64	1.0197±0.0039	3.82 ±0.21	1.0204±0.0010	1.0063±0.0006

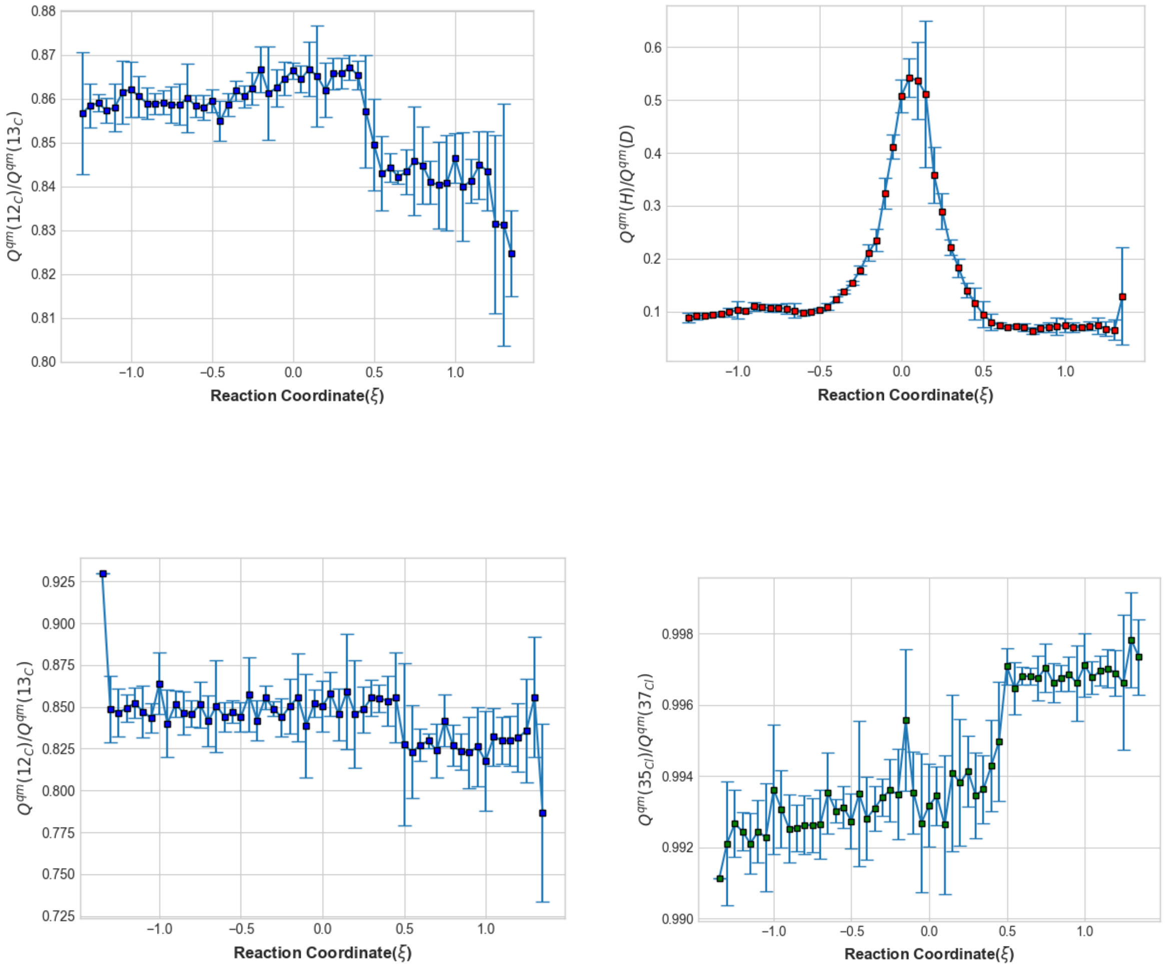

Figure S3. The ratio of quantum mechanical partition functions (equivalent to the free energy difference) between ^{12}C and ^{13}C for atom C_1 (top left) ,between H and D for atom H_1 (top right), between ^{12}C and ^{13}C for atom C_2 (top left) and between ^{35}Cl and ^{37}Cl for atom Cl_1 obtained using QM(PM3)/MM potential and 64 beads.

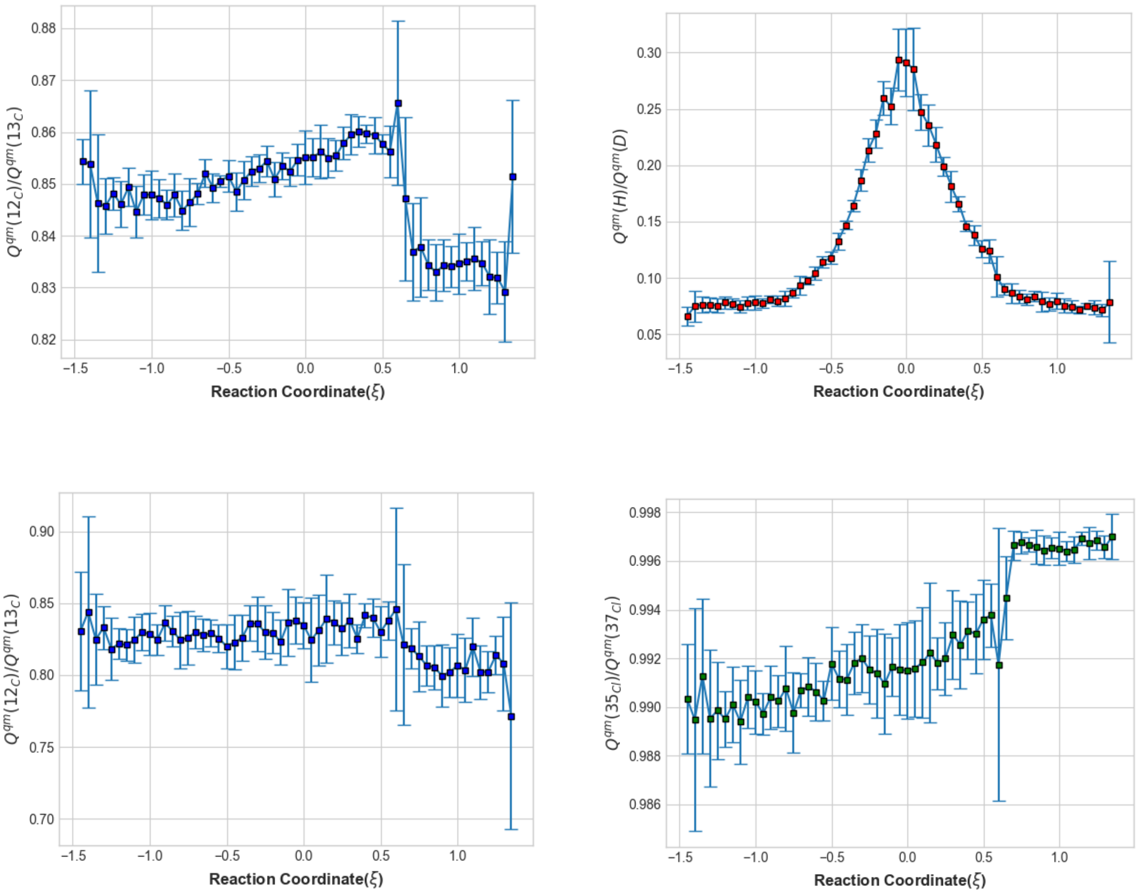

Figure S4. The ratio of quantum mechanical partition functions (equivalent to the free energy difference) between ^{12}C and ^{13}C for atom C_1 (top left) ,between H and D for atom H_1 (top right), between ^{12}C and ^{13}C for atom C_2 (top left) and between ^{35}Cl and ^{37}Cl for atom Cl_1 obtained using QM(AM1)/MM potential and 64 beads.

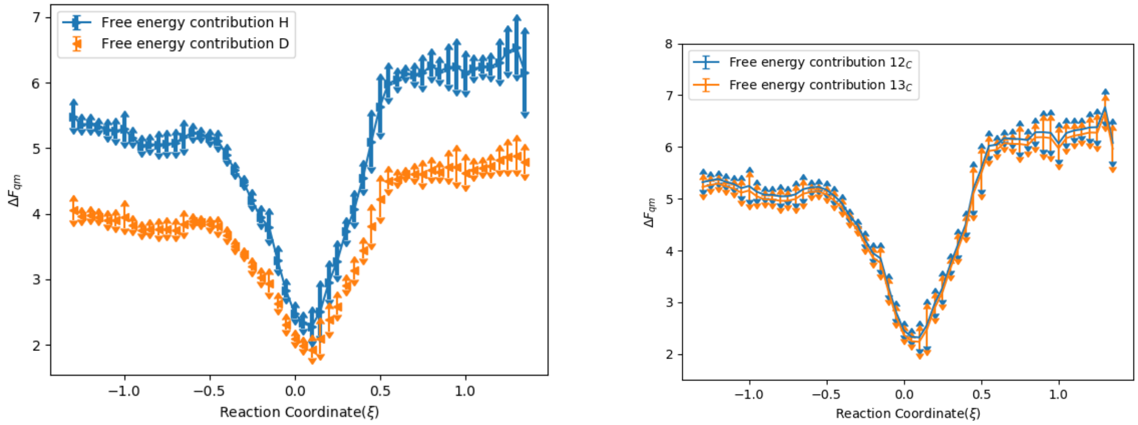

Figure S5. The free energy contribution for H (blue) and D (orange) for atom H_1 (left) and the free energy contribution for ^{12}C (blue) and ^{13}C (orange) for atom C_1 obtained using QM(PM3)/MM potential and 64 beads.

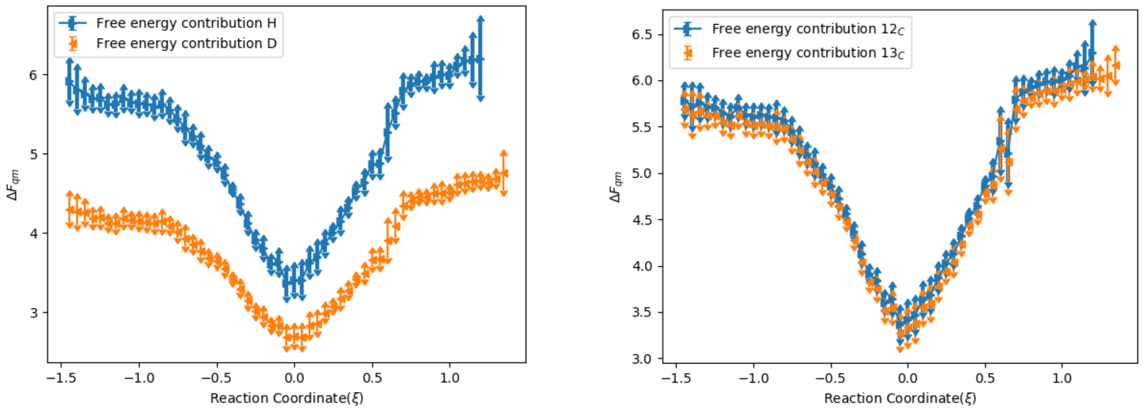

Figure S6. The free energy contribution for H (blue) and D (orange) for atom H_1 (left) and the free energy contribution for ^{12}C (blue) and ^{13}C (orange) for atom C_1 obtained using QM(AM1)/MM potential and 64 beads.

Bare

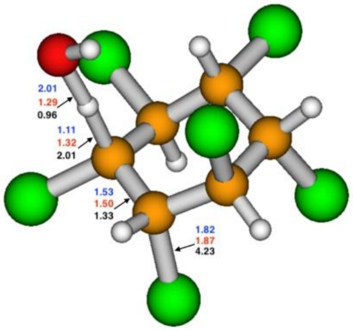

1W_OH

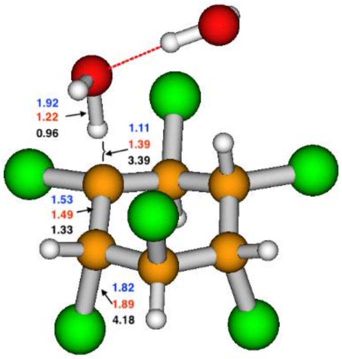

1W_OH_1W_Cl

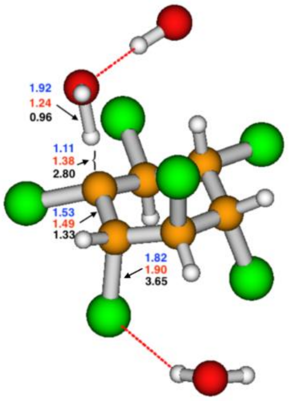

2W_OH

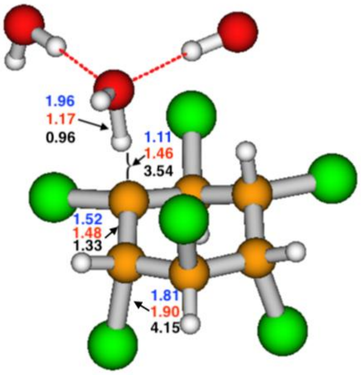

2W_OH_2W_Cl

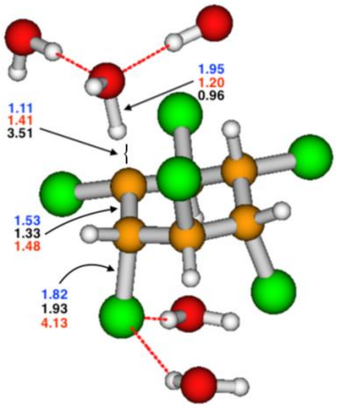

3W_OH

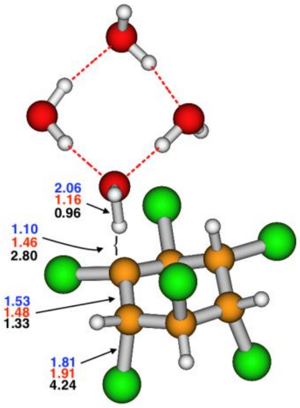

3W_OH_3W_Cl

3W_OH_4W_Cl

3W_OH_5W_Cl

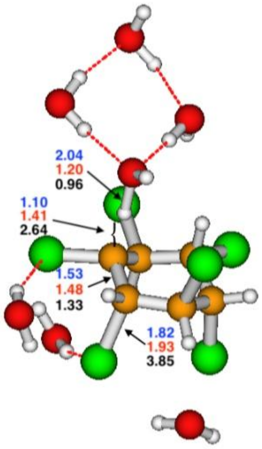

4W_OH

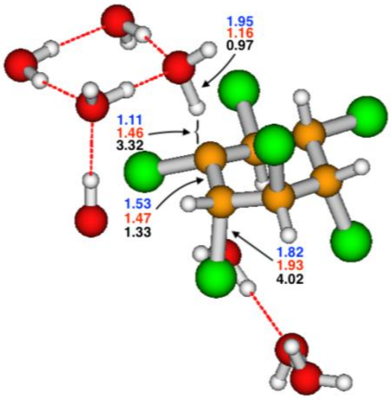

4W_OH_4W_Cl

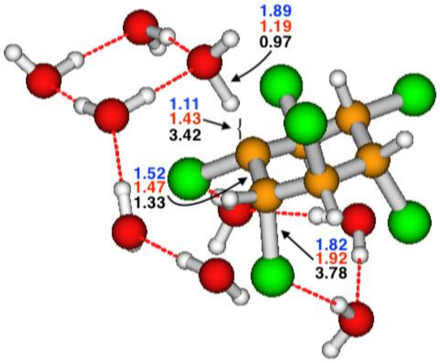

4W_OH_5W_Cl

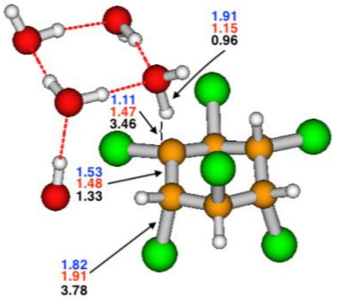

4W_OH_6W_Cl

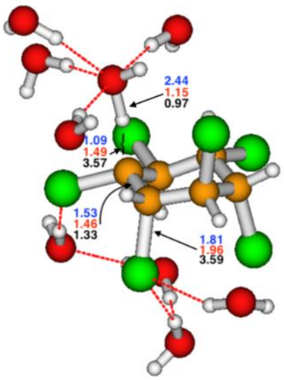

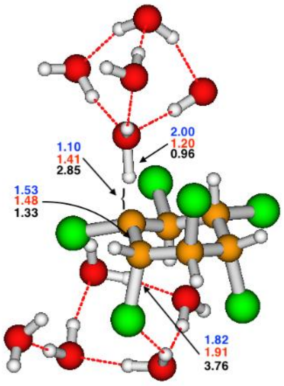

5W_OH

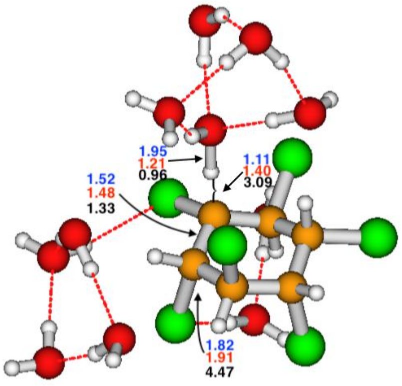

QM(AM1)/MM

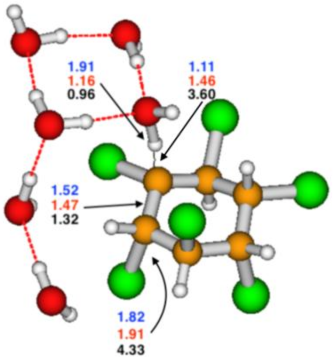

QM(PM3)/MM

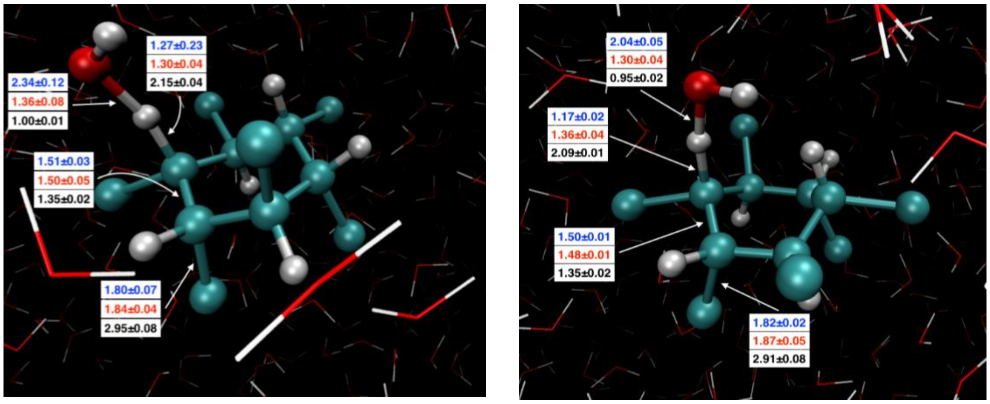

H_2O_2W

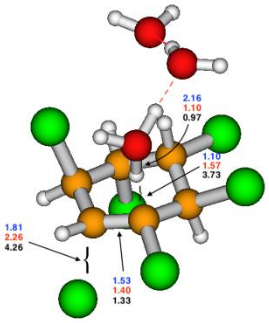

Figure S7. Key geometrical parameters for all cluster and QM/MM models for position 1 of the γ-HCH isomer (blue for reactants, red for transition states, and black for products).

Table S6a. Merz-Kollman atomic charges (in a.u.) on the key atoms at each stationary point for the bare model and microsolvation models in which only hydroxyl anion was hydrated obtained at the ωB97xD/6-311+G(d,p)/PCM level of theory.

ESP	Bare model			1W_OH		
	R	TS	P	R	TS	P
**C1**	-0.2	-0.2	0.1	-0.6	-0.1	0.1
**H1**	0.4	0.5	0.4	0.7	0.5	0.4
**C2**	-0.3	-0.5	0.0	-0.4	-0.3	0.0
**Cl2**	-0.1	-0.1	-1.0	0.0	-0.2	-1.0
**O**	-1.4	-1.3	-0.9	-1.4	-1.3	-0.9
	**2W_OH**			**3W_OH**		
	R	TS	P	R	TS	P
**C1**	-0.5	0.0	0.0	-0.5	-0.2	0.0
**H1**	0.6	0.2	0.3	0.6	0.5	0.3
**C2**	-0.4	-0.1	0.0	-0.6	-0.3	-0.1
**Cl2**	0.0	-0.2	-1.0	0.0	-0.2	-1.0
**O**	-1.3	-0.8	-0.5	-1.2	-1.1	-0.6
	**4W_OH**			**5W_OH**		
	R	TS	P	R	TS	P
**C1**	-0.6	-0.2	0.1	-0.7	-0.1	0.3
**H1**	0.7	0.5	0.4	0.8	0.4	0.4
**C2**	-0.6	-0.4	0.1	-0.4	-0.3	-0.1
**Cl2**	0.0	-0.2	-1.0	0.0	-0.2	-1.0
**O**	-1.2	-1.0	-0.8	-1.4	-1.0	-0.9

Table S6b. Merz-Kollamn atomic charges (in a.u.) on the key atoms at each stationary point for the microsolvation models in which the hydroxyl and chloride anions were hydrated obtained at the ωB97xD/6-311+G(d,p)/PCM level of theory.

	**1W_OH_1W_Cl**			**2W_OH_2W_Cl**		
	R	TS	P	R	TS	P
**C1**	-0.8	-0.4	0.1	-0.7	-0.4	-0.1
**H1**	0.8	0.6	0.4	0.7	0.4	0.3
**C2**	-0.1	0.0	0.1	0.0	0.3	0.2
**Cl2**	0.0	-0.2	-0.9	-0.1	-0.3	-0.9
**O**	-1.5	-1.3	-0.9	-0.1	-0.8	-0.6

	**3W_OH_3W_Cl**			**3W_OH_4W_Cl**			**3W_OH_5W_Cl**		
	R	TS	P	R	TS	P	R	TS	P
**C1**	-0.7	-0.9	-0.2	-0.6	0.4	1.0	-2.1	-0.5	0.0
**H1**	0.8	0.8	0.3	0.6	0.1	0.3	1.4	0.6	0.4
**C2**	-1.0	0.2	0.2	-0.4	-0.2	-0.3	-0.5	-0.6	0.9
**Cl2**	0.1	-0.1	-0.9	-0.1	-0.2	-0.9	0.0	-0.1	-0.9
**O**	-1.4	-1.1	-0.6	-1.0	-0.6	-0.6	-1.3	-1.0	-0.9

	**4W_OH_4W_Cl**			**4W_OH_5W_Cl**			**4W_OH_6W_Cl**		
	R	TS	P	R	TS	P	R	TS	P
**C1**	-1.1	-0.7	0.0	-0.9	-0.6	0.0	-0.4	0.3	0.0
**H1**	1.2	0.7	0.2	0.5	0.5	0.3	0.5	0.4	0.4
**C2**	0.0	0.2	0.4	0.1	-0.1	0.2	-0.7	-1.0	0.5
**Cl2**	-0.1	-0.3	-0.9	-0.2	-0.3	-0.9	0.1	0.0	-0.9
**O**	-1.0	-0.8	-0.1	-1.2	-1.1	-0.7	-1.2	-1.1	-0.6

Table S7. Key geometrical parameters of transition states located using different models of dehydrochlorination of γ-HCH. The shaded areas show similarities between the models.

Model	C1-H1	H1···base[a]	C1-C2	C2-Cl2
H_2O_2W	1.57	1.10	1.40	2.26
3W_OH	1.38	1.24	1.49	1.90
4W_OH-4W_Cl	1.46	1.16	1.47	1.93
QM(AM1)/MM	1.30	1.36	1.50	1.84
LinA[b]	1.54	1.19	1.48	1.95

[a] – it is water, hydroxyl group, and His73 in the case of H_2O_2W, alkaline hydrolysis, and LinA models, respectively
[b] – Ref. 7

Minimum free energy path using the string method

Similarly to the simulations in CHARMM described in the main article the TIP3P water model and a hybrid QM/MM scheme with the PM3 and AM1 Hamiltonians were used. MD simulations were performed with a Langevin thermostat at 300 K, Velocity Verlet integrator [1,2] and periodic boundary conditions with Particle Mesh Ewald [3,4] to treat long-range electrostatic interactions. Replica exchange was applied during both the string optimization and the US with exchange attempts performed every 100 fs. Three collective variables (CVs) were defined to monitor the elimination of the H/Cl pair from the γ-HCH molecule by the hydroxyl anion; the C1-H1, H1-O, and C2-Cl2 interatomic distances (Scheme 1). 50 nodes were used to follow the reaction progress to the products. In order to keep the abstracted hydrogen on the path we used the biasing potential, Vb(z) and increased the mass of H1 to 2. The resulting QM(PM3)/MM and QM(AM1)/MM PMFs were subsequently corrected using the higher level of theory for the QM part of the system. For this purpose the path was optimized using the parameters resulted from the string calculations such as force constants, nodes positions, etc. This optimization was performed using either the PM3/TIP3P or AM1/TIP3P combination, then resulting structures were subjected to the single-point energy calculations at the ωB97xD/6-311+G(d,p) level of theory carried out using the Gaussian interface with the Amber package.

Determination of the experimental isotope enrichment factors of γ-HCH

Hydrolysis of γ-HCH was carried out in 100 mM NaOH-H_3BO_3 buffer at 30 °C (pH 10,) as batch experiments. The enzymatic degradation of γ-HCH by LinA was performed in TRIS buffer (pH 7.5) at 10°C and 120 rpm shaking. The reactions were stopped at different time intervals and the remaining γ-HCH was extracted for concentration and stable carbon, hydrogen and chlorine isotope analysis. The analytical methods have been described in details elsewhere [5,6], here only basic information is provided.

The concentration of γ-HCH throughout the study was determined using an Agilent 6890 series GC (Agilent Technologies, USA) equipped with a flame ionization detector (FID). A HP-5 column (30 m × 320 μm × 0.25 μm, Agilent 19091J-413, USA) was applied for the separation with helium as the carrier gas at a flow of 1 mL min^{-1}.

Carbon isotope composition ($\delta^{13}C$) was analyzed using a gas chromatograph-combustion-isotope ratio mass spectrometer (GC-C-IRMS) system, where a GC (7890A, Agilent Technologies, USA) was coupled via a ConFlo IV interface (Thermo Fisher Scientific, Germany) to a MAT 253 IRMS system (Thermo Fisher Scientific, Germany). Samples were injected in splitless mode at 250 °C and then separated on a Zebron ZB1 column (60 m × 0.32 mm × 1 μm; Phenomenex, Germany) under a constant carrier gas flow of 1.5 mL min^{-1}. After separation, compounds were converted to CO_2 in the combustion reactor operating at 1000 °C. Samples were analyzed in triplicates. Corresponding analytical precision for $\delta^{13}C$ was below ±0.5‰.

Hydrogen isotope compositions ($\delta^{2}H$) were analyzed via a gas chromatography-chromium based high temperature conversion-isotope ratio mass spectrometry (GC–Cr/HTC–IRMS) system. Cr/HTC was applied to make use of the combination of high temperature conversion and reduction of hot elemental chromium at 1200 °C in order to scavenge Cl as chromium salt, so that H_2 can be released into the carrier stream and subsequently isotopically analyzed by the IRMS. The GC parameters were the same as described for the $\delta^{13}C$ measurement. Samples were analyzed in triplicates. Corresponding analytical precision for $\delta^{2}H$ was below ±5‰.

Chlorine isotope composition ($\delta^{37}Cl$) was determined online using gas chromatography coupled with multiple-collector inductively coupled plasma mass spectrometry (GC-MC-ICPMS) system. Samples were injected with a split ratio of 1:10 and a constant carrier gas flow of 2 mL min^{-1}. Once separated, the analyte was directed to the ICP torch via a Thermo Elemental Transferline AE2080 (Aquitaine Electronique, France). The MC-ICPMS plasma was operating at dry plasma conditions, reducing unfavorable protonation effects. The chlorine isotopes were analyzed at mass 35 and 37 ($^{35}Cl^{+}$, $^{37}Cl^{+}$) at low resolution mode (m/Δm = 300). Samples were generally analyzed in triplicates, with an analytical precision usually below ±0.3‰ for compound-specific analysis of mixtures.

The isotope composition of element (E) is reported as δ notation in parts per thousand (‰) and expressed as the deviation from international standards according to the following equation:

$$\delta E_{sample} = (R_{sample}/R_{standard}) - 1$$

Where R indicates the isotope ratio of $^{13}C/^{12}C$, $^{2}H/^{1}H$ or $^{37}Cl/^{35}Cl$. International standards for ^{13}C, ^{2}H and ^{13}Cl are Vienna Pee Dee Belemnite (VPDB), Vienna Standard Mean Ocean Water (VSMOW) and Standard Mean Ocean Chloride (SMOC), respectively.

The isotope enrichment factor (ε) was determined from the logarithmic form of the Rayleigh equation as following:

$$\ln\left(\frac{\delta E_t + 1}{\delta E_0 + 1}\right) = \varepsilon \times \ln\left(\frac{C_t}{C_0}\right)$$

Where δE_t and δE_0 are the isotopic signatures of the compound for the element E at a given time t and at the beginning of the reaction; while C_t/C_0 is the fraction of the remaining compound.

References

[1] Verlet, L. "Computer 'Experiments' on Classical Fluids. I. Thermodynamical Properties of Lennard−Jones Molecules". *Phys. Rev.* **1967**, *159*, 98–103.

[2] Swope, W.C.; Andersen, H.C.; Berens, P.H.; Wilson, K.R. "A computer simulation method for the calculation of equilibrium constants for the formation of physical clusters of molecules: Application to small water clusters." *J. Chem. Phys.* **1982**, *76*, 648 (Appendix).
[3] Ewald, P. "Die Berechnung optischer und elektrostatischer Gitterpotentiale" *Ann. Phys.* **1921**, *64*, 253.
[4] Essmann, U., Perera, L., Berkowitz, M.L., Darden, T., Lee, H., Pedersen, L.G., "A smooth particle mesh Ewald method" *J. Chem. Phys.* **1995**, *103*, 8577-8593.
[5] Wu, L.; Moses, S.; Liu, Y.; Renpenning, J.; Richnow, H.H. "A concept for studying the transformation reaction of hexachlorocyclohexanes in food webs using multi-element compound-specific isotope analysis". *Anal. Chim. Acta.* **2019**, 1064, 56-64
[6] Wu, L.; Liu, Y.; Liu, X.; Bajaj, A.; Gaur, M.; Lal, R.; Richnow, H. H., "Isotope fractionation approach for characterization of the reactive transport processes governing the fate of hexachlorocyclohexanes at a contaminated site in India". *Environ. Int.* **2019**,132,105036.
[7] Manna, R.N.; Zinovjev, K.; Tuñón, I.; Dybala-Defratyka, A. "Dehydrochlorination of Hexachlorocyclohexanes Catalyzed by the LinA Dehydrohalogenase. A QM/MM Study" *J. Phys. Chem. B* **2015**, *119*, 15100.
